# Cardiovascular outcomes improve in children with renovascular hypertension following endovascular and surgical interventions

**DOI:** 10.1007/s00467-023-06123-5

**Published:** 2023-09-02

**Authors:** Emily C. G. Redhead, Alicia Paessler, Zainab Arslan, Premal Patel, Kishore Minhas, Colin Forman, Paolo Hollis, Sebastiano Lava, Florin Ionescu, Devi Manuel, Samiran Ray, Nicos Kessaris, Alessandro Giardini, Vineetha Ratnamma, Nadine Dobby, Kjell Tullus, Jacob Simmonds, Jelena Stojanovic

**Affiliations:** 1 University College London Great Ormond Street Institute of Child Health, London, UK; 2 Great Ormond Street Hospital for Children NHS Foundation Trust, Renal Unit, Level 7, Southwood Building, Great Ormond Street, London, WC1N 3JH UK

**Keywords:** Paediatric, Hypertension, Cardiac, Angioplasty

## Abstract

**Background:**

Renovascular hypertension (RenoVH) is a cause of hypertension in children. A common cause of RenoVH is renal artery stenosis which acts by reducing blood supply to renal parenchyma and activating the renin–angiotensin–aldosterone axis, often leading to cardiac remodelling. This longitudinal observational study aims to describe occurrence of cardiovascular changes secondary to RenoVH and also any improvement in cardiac remodelling after successful endovascular and/or surgical intervention.

**Methods:**

All patients with RenoVH referred to our centre, who received ≥ 1 endovascular intervention (some had also undergone surgical interventions) were included. Data were collected by retrospective database review over a 22-year period. We assessed oscillometric blood pressure and eight echocardiographic parameters pre- and post-intervention.

**Results:**

One hundred fifty-two patients met inclusion criteria and had on average two endovascular interventions; of these children, six presented in heart failure. Blood pressure (BP) control was achieved by 54.4% of patients post-intervention. Average *z*-scores improved in interventricular septal thickness in diastole (IVSD), posterior Wall thickness in diastole (PWD) and fractional shortening (FS); left ventricular mass index (LVMI) and relative wall thickness (RWT) also improved. PWD saw the greatest reduction in mean difference in children with abnormal (*z*-score reduction 0.25, *p* < 0.001) and severely abnormal (*z*-score reduction 0.23, *p* < 0.001) *z*-scores between pre- and post-intervention echocardiograms. Almost half (45.9%) had reduction in prescribed antihypertensive medications, and 21.3% could discontinue all antihypertensive therapy.

**Conclusions:**

Our study reports improvement in cardiac outcomes after endovascular + / − surgical interventions. This is evidenced by BP control, and echocardiogram changes in which almost half achieved normalisation in systolic BP readings and reduction in the number of children with abnormal echocardiographic parameters.

**Graphical abstract:**

**Figure:**
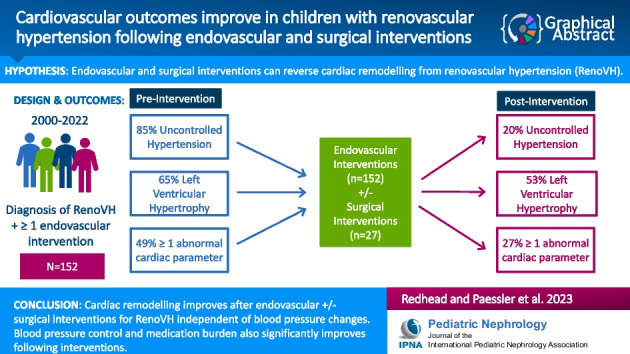
A higher resolution version of the Graphical abstract is available as Supplementary information.Supplementary information

**Supplementary Information:**

The online version contains supplementary material available at 10.1007/s00467-023-06123-5.

## Introduction

Renovascular hypertension (RenoVH) is characterised by severe hypertension (Systolic BP > 99th centile) which is most commonly due to renal artery stenoses [1]. Subsequent renal hypoperfusion and activation of the renin–angiotensin–aldosterone system exacerbate the increased BP, often leading to target organ injury [1]. Typical cardiac manifestations of hypertension in children and adolescents are left ventricular hypertrophy (LVH) with reduced left ventricular systolic and diastolic function [2].

Up to 5% of hypertension (HTN) in children is caused by RenoVH [3]. In current clinical practice, treatment interventions range from pharmacological management to endovascular and surgical interventions, depending on the clinical status and underlying lesion of each patient [4]. The body of current literature on paediatric RenoVH includes information on the effectiveness of treatment in controlling blood pressure [3], while information on the correlation between treatment and cardiovascular outcomes is missing. Furthermore, the severity of cardiac involvement in children with RenoVH and the number of (percutaneous or surgical) interventions required to improve cardiovascular involvement are also not known.

This study reports the echocardiographic measures and function at both the time of diagnosis and post-intervention and addresses the number of required endovascular or surgical interventions. This study aims to investigate whether there is an improvement in the remodelling or function of the heart after children undergo surgical or endovascular intervention to treat RenoVH, as well as how many interventions were required to achieve these cardiovascular changes. We hypothesised that interventions may help to reverse some of the cardiac remodelling seen in children with RenoVH and improve cardiac function.

## Materials and methods

### Study design and setting

This longitudinal observational retrospective cohort study was conducted at Great Ormond Street Hospital (GOSH) and included patients diagnosed with RenoVH over a 22-year period (2000–2022).

Ethics approval for this study was waived by our local centre as all patient information was pseudoanonymised during data collection and fully anonymised prior to analysis and was only accessed by members of the study team.

### Study population

Our inclusion criteria were the following:

- Diagnosis of RenoVH on angiography
- At least one endovascular intervention during childhood
- Treatment for RenoVH occurred at our centre between 1999 and 2022
- Digital copies of pre- and post-procedure echocardiograms and procedure details available

Patients were identified using our RenoVH database; each child diagnosed with RenoVH and treated at our centre is added to this database. At our centre, the indication for interventions was confirmed diagnosis of RenoVH and uncontrolled hypertension despite maximum dose of 2 or more antihypertensives. Endovascular interventions included percutaneous transluminal angioplasty, cutting balloon angioplasty, stenting or ethanol ablation. Further 27 participants in whom endovascular procedures could not achieve sustained BP control also underwent surgical interventions.

### Definitions

- Blood pressure centiles were defined as per the American Academy of Paediatrics Clinical Practice Guidelines [2].
- Surgical interventions in this manuscript refer to a range of operations used to treat RenoVH that include aortic bypass, in situ arterial re-implantation, nephrectomy and auto-transplant.
- Interventricular septal thickness in diastole (IVSD) is measured perpendicular to the interventricular septum wall at end-diastole.
- Posterior wall thickness in diastole (PWD) is a measured perpendicular to the posterior wall of the left ventricle at end-diastole.
- Left ventricular internal diameter in diastole (LVIDD) is a measurement of the internal dimension of the left ventricle at end-diastole and is measured perpendicular to the long axis of the left ventricle at the level of the mitral valve leaflet tips.
- Left ventricular internal diameter in systole (LVIDS) is measured in the same way as the LVIDD, but the measurement is taken at end-systole.
- Left ventricular mass (LVM) was calculated using the Devereux formula [5] and is a measure of the total mass of the left ventricle:
- Devereux formula: 0.80 (1.04 (LVIDD + PWD + IVSD)^3^ − (LVIDD)^3^) [5]
- Left ventricular mass index (LVMI) is the LVM indexed to patient body surface area:
- LVMI = LVM/BSA
- where BSA = 0.007184 × (weight)^0.425^ × (height)^0.725^
- Relative wall thickness (RWT) measures how thick the ventricular walls are in diastole relative to the internal diameter of the left ventricle during diastole:
- RWT = (IVSD + PWD)/LVIDD
- Fractional shortening (FS) was calculated using the LVIDD and LVIDS measurements, and this is a measure of how well the left ventricle can contract itself:
- FS = (LVIDD − LVIDS)/LVIDD × 100%
- Left ventricular ejection fraction (LVEF) was calculated using the Teicholz method and is an assessment of global systolic function of the left ventricle:
- LVEF = (LVEDV − LVESV)/LVEDV × 100%
- LVEDV = Left ventricular end-diastolic volume = 7/(2.4 + LVIDD) × LVIDD^3^
- LVESV = Left ventricular end-systolic volume = 7/(2.4 + LVIDS) × LVIDS^3^
- For echocardiographic measurements (IVSD, PWD, LVIDD and LVIDS), a *z*-score ≥  + 3 or ≤  − 3 was considered severely abnormal, ≥  + 2 or ≤  − 2 as abnormal and between >  − 2 and <  + 2 as normal.
- An FS ≤ 20% was considered severely abnormal, ≤ 28% as abnormal and > 28% as normal.
- LVEF < 56% was considered abnormal.
- LVMI was considered normal if < 38.6 g/m^2.7^ with any patients exceeding that defined as having left ventricular hypertrophy (LVH) [6].
- RWT was considered normal if < 0.42.
- Patients were also classified in terms of their cardiac geometry as follows:
- Normal geometry (normal LVMI + normal RWT)
- Concentric hypertrophy (increased LVMI and increased RWT)
- Eccentric hypertrophy (increased LVMI and normal RWT)
- Concentric remodelling (normal LVMI and increased RWT)
- Estimated glomerular filtration rate (eGFR) was calculated pre- and post-intervention using the modified Schwartz equation [7] (CKiD), in which the constant *k* = 36.2.

### Characterization of the study subjects

Data for each patient were sourced from the earliest available record until their last follow-up or end of data collection, up to June 2022. Variables that were lacking in electronic health record systems were noted as missing.

We collected five subsets of data for each patient at two time points: first recorded value (before intervention) and last recorded value (after intervention) in the following categories: patient demographics (age, sex, country of origin, underlying syndrome), antihypertensive drug(s), cardiovascular (systolic BP centile, daytime systolic BP load (percentage of readings greater than a threshold value) [2]), renal (surgical interventions, kidney function) and endovascular intervention (type, location).

The blood pressure data were collected through two methods at various time points. Systolic BP was always recorded from an office BP, as not all patients, particularly those who presented earlier during the study period, underwent ambulatory blood pressure measurement (ABPM), so this allowed the same variable to be collected for all participants. Patients who did undergo ABPM had their daytime systolic BP load recorded from their ABPM report pre- and post-intervention. At the time of all recorded pre-intervention blood pressure values, all patients were on antihypertensive therapy as all patients referred to our tertiary centre were already on pharmacological management for their hypertension. Raw systolic blood pressure values were then classified as age-, sex- and height-specific centiles [2].

We documented data from every first echocardiogram which was done at the first presentation pre-procedure and patients’ last echocardiograms which were always > 6 months post-endovascular intervention. This allowed enough time for a potential echocardiographic change to be evident and detectable post-intervention. At the time of the first echocardiogram, all children were receiving maximum tolerated antihypertensive therapy but had not undergone any endovascular or surgical procedures yet. All echocardiograms were re-reviewed offline by four cardiologists solely for the purpose of the study. The reviewers were blinded to the nature of the study and goals of the study as well as the identity and clinical status of the patients at the time of echocardiogram. They recorded measurements of IVSD, PWD, LVIDD and LVIDS for each echocardiogram. Aside from re-measuring all the parameters, they also re-calculated the *z*-scores. The absolute values were inputted into Boston Children’s *z*-score calculator with the patient’s age, height, weight and sex at the time of the correlating echocardiogram to produce the corresponding *z*-scores. The Boston Children’s *z*-score calculator uses data based on over 12 years of data collection from healthy children and is used to calculate *z*-scores for echocardiographic measures based on a child’s age, height, weight and sex. LVM, LVMI, RWT, LVEF and FS were also then calculated (as described above). Measurements were taken under the following confines: 2-dimensional view (if this was not available, then M-mode was used) and along the parasternal short axis (if this was not available, then the parasternal long axis was used). *Z*-scores (Boston Children’s) were calculated based on sex, weight and height. For consistency (pairwise comparison), it was ensured that the same view was used for all measurements of each individual patient. Unfortunately, diastolic functional assessment was not available and so was not reported in this study.

### Outcomes and statistical methods

The primary outcome variables included the changes in cardiac dimensions and function on echocardiograms before and after endovascular + / − surgical interventions. The secondary outcomes included BP control, preservation of kidney function and number of prescribed antihypertensive medications.

All statistical analysis was carried out on IBM Statistical Package for Social Sciences (SPSS) software version 28.0.1.0 (Chicago, IL, USA) ^8^. Before statistical testing, normality assessments with Shapiro–Wilk test were performed in which *p*-values < 0.05 indicated normally distributed data.

Descriptive statistics summarised the variables within the dataset. To compare mean differences between subgroups at different time points (before and after intervention or between first and last recorded values), continuous data were analysed with the paired sample *t*-test for parametric data or the Wilcoxon matched-pair signed-rank tests for non-parametric data. Proportions were analysed by means of the chi-square tests. All results are reported with 95% confidence intervals, with a *p*-value < 0.05 indicating statistical significance. Multivariate linear regression was also carried out to investigate the possibility of any confounding relationships between blood pressure and antihypertensive medication with cardiac changes (changes in LVMI). The covariates included SBP centiles, age, presence of MAS, number of antihypertensive medications, types of antihypertensive medications, number of interventions and if the patient underwent surgical interventions.

## Results

### Participants

An initial cohort of 172 children who were referred to our centre and underwent endovascular interventions/surgery were identified over the study period. However, 20 patients did not have digital records of their endovascular interventions, so these were excluded which resulted in a final cohort of 152 patients.

Over half the patients (*n* = 95, 62.5%) were male, and the median age at the time of referral was 6.9 years (IQR 3–9). The majority (*n* = 99, 65.1%) of patients resided in the UK at the time of the referral, with others residing in 20 other countries worldwide. Less than half (*n* = 43, 28.3%) of patients had an underlying diagnosis, with the most common being neurofibromatosis type 1 (NF1) (*n* = 22, 14.5%). Overall, 59 children (38.8%) presented with middle aortic syndrome (MAS). Almost half (*n* = 61, 42%) of patients had unilateral lesions, 74 (51%) had bilateral lesions and 9 patients (6%) had disease in accessory renal arteries but not the main renal arteries. See Supplementary File 1 for full patient demographics.

All patients had at least one endovascular intervention, and 27 participants (17.7%) also received a surgical intervention. The surgical interventions included unilateral (*n* = 15, 9%) and bilateral nephrectomies (*n* = 2, 1%), unilateral (*n* = 10, 6%) and bilateral auto-transplant (*n* = 1, 1%) and living donor (*n* = 1, 1%) and deceased donor (*n* = 4, 2%) kidney transplants. The median number of interventions performed in the study was 2 (IQR 1–4). The median overall follow-up time from the date of referral was 3.8 years, with the median time between first (pre-procedure) and last (post-procedure) echocardiogram being 2.6 years (IQR 1.1–6.7). At last follow-up, the mean patient age was 9.5 years, with 16 patients (11%) being older than 18 years at last follow-up. The last echocardiograms were at least 6 months post-procedure to allow enough time for cardiac remodelling to occur.

### Blood pressure

**Fig. 1:**
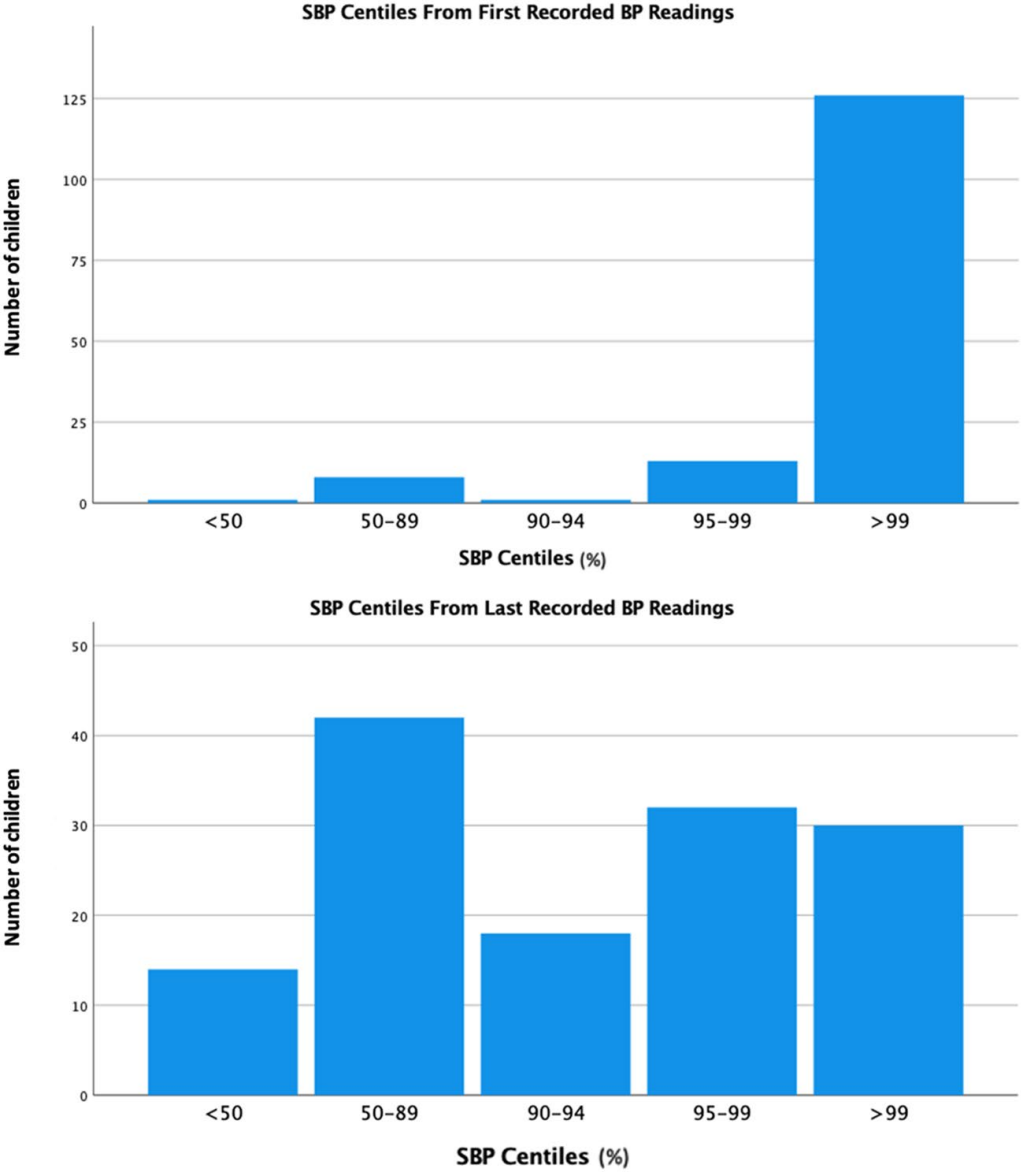
Bar graphs displaying the number of patients within each systolic blood pressure centile based on each patient’s first (*n* = 152, top graph) and last (*n* = 136, bottom graph) systolic blood pressure recording. SBP, systolic blood pressure; BP, blood pressure

As displayed in Fig. 1, 139 patients (91.4%) had SBP readings above the 95th centile, and 129 patients (84.6%) had a SBP above the 99th centile at presentation. By last follow-up (median follow-up time between first and last BP was 3.8 years), the number of patients with SBP above the 95th and 99th centile significantly decreased to 69 patients (45.6%) and 30 patients (19.7%), respectively (*p* < 0.001). Overall, 68 patients (45%) had pre- and post-procedure ABPM measurements which allowed assessment of daytime average SBP load. The mean daytime average SBP load also significantly reduced post-intervention from 60.4 to 44.4 (*p* = 0.02).

### Medical treatment

Our centre’s approach to antihypertensive therapy involves calcium channel blockers first-line followed by beta-blockers, then alpha-blockers, then diuretics, and if patients still had uncontrolled hypertension, the use of ACE inhibitors and angiotensin-receptor blockers was carefully considered. The maximum dose of each drug was tried before the addition of another antihypertensive.

At referral to our centre, 140 patients (91.9%) were receiving antihypertensive therapy with 77 patients (50.7%) requiring 3 or more antihypertensives. This significantly reduced to 120 patients (78.7%) (*p* < 0.01) still needing antihypertensive therapy and 58 patients (38.2%) (*p* = 0.02) requiring 3 or more antihypertensive post-procedure. Overall, 70 patients (45.9%) had seen a reduction in the number of prescribed antihypertensive post-intervention. The differences in cardiac outcomes based on the type of antihypertensives used were not investigated in this study.

### Cardiac parameters

All first echocardiograms were carried out prior to any interventions, and all last echocardiograms were performed at least 6 months post-intervention with a median time interval of 2.6 years. LVMI significantly decreased by a mean of 18.3 g/m^2.7^ from pre- to post-intervention (*p* < 0.01), and the number of patients with LVH also decreased from 99 patients (65.4%) to 80 patients (53.1%) although this was not statistically significant. RWT also significantly decreased by 0.04 post-intervention (*p* = 0.01). LVEF and FS improved post-intervention although not to a statistically significant degree. There was also a significant improvement in average IVSD and PWD (*p* = 0.01 and *p* < 0.01) as demonstrated by a reduction in *z*-scores. The mean *z*-score for LVIDD on last echocardiograms remained within normal limits but increased marginally by 0.08 (− 0.13 to − 0.05), as shown in Table 1.

**Table 1:** Average *z*-scores from first (pre-intervention) and last (post-intervention) echocardiograms

Echo	IVSD *z*-score average	PWD *z*-score average	LVIDD *z*-score average	LVMI (g/m^2.7^) average	RWT average	LVEF (%) average	FS (%) average
Pre-intervention	+ 1.57 (1.10 to 2.04)	+ 2.16 (1.65 to 2.67)	− 0.13 (− 0.56 to 0.30)	55.86 (47.74 to 63.98)	0.40 (0.38 to 0.42)	59.4 (47.2 to 61.6)	36.29 (34.76 to 37.81)
Post-intervention	+ 1.05 (− 1.44 to 3.54)	+ 1.07 (− 1.45 to 3.57)	− 0.05 (− 0.59 to 0.49)	48.46 (39.58 to 57.34)	0.37 (0.35 to 0.39)	61.03 (58.63 to 63.43)	37.13 (35.36 to 38.90)
Mean difference 2-sided *p*-value	**0.016**	** < 0.001**	0.65	** < 0.001**	**0.01**	0.16	0.31

Averages are expressed as mean (95% CI) values. Paired *t*-test, significance level *p* < 0.05

*Echo* echocardiogram, *IVSD* interventricular septal thickness in diastole, *PWD* posterior wall thickness in diastole, *LVIDD* left ventricular internal diameter during end-diastole, *LVMI* left ventricular mass index, *RWT* relative wall thickness, *LVEF* left ventricular ejection fraction, *FS* fractional shortening

Values in bold represent statistically significant changes

**Fig. 2:**
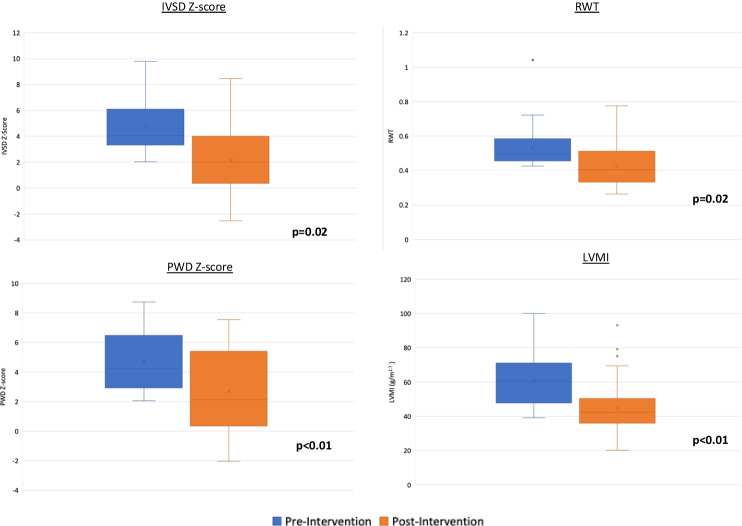
Multiple box plots displaying the significant change in IVSD and PWD *z*-scores, LVMI and RWT post-intervention in those who presented with abnormal parameters pre-intervention. Other cardiac parameters not included in this figure did not change significantly; these included LVIDD, LVEF and FS. IVSD, interventricular septal thickness in diastole; PWD, posterior wall thickness in diastole; LVMI, left ventricular mass index; RWT, relative wall thickness; LVIDD, left ventricular internal diameter during diastole; LVEF, left ventricular ejection fraction; FS, fractional shortening

Almost half of the patients (*n* = 60, 39.5%) had at least one abnormal echocardiographic parameter on either their first or last echocardiogram, and of those, 47 children (30.9%) had at least one severely abnormal parameter. As exhibited in Fig. 2, there was a trend of improvement in all cardiac parameters whereby wall thickness *z*-scores decreased, LVMI decreased and FS increased over time, indicating an echocardiographic improvement in those who presented with abnormal echocardiographic parameters.

A subgroup analysis focussing only on patients with at least one abnormal or severely abnormal echocardiographic parameter was performed, comparing values at baseline and at their last available follow-up. IVSD (*p* ≤ 0.04) and PWD (*p* ≤ 0.001) decreased significantly in both subgroups, as shown in Table 2. The proportion of patients with at least moderately impaired contractility (FS ≤ 20) fell from 7 patients (4.8%) to 4 patients (2.5%), and those with LVH fell from 99 patients (65.4%) to 80 patients (53.1%), but these were not significant (*p* = 0.36 and *p* = 0.08, respectively).

**Table 2:** Difference in proportion of patients with abnormal or severely abnormal cardiac parameters on pre- and post-intervention echocardiograms

Parameter	Proportion of patients on pre-intervention echocardiogram	Proportion of patients on post-intervention echocardiogram	Two-sided *p*-value
IVSD ≥ + 2	36%	31.3%	**0.04**
PWD ≥ + 2	44.8%	30%	** < 0.001**
LVIDD ≥ + 2	12.8%	11.3%	0.46
LVMI > 38.6	65.4%	53.1%	0.08
RWT > 0.42	33.6%	22.8%	0.10
LVEF < 56%	33.6%	30.4%	0.63
FS ≤ 28%	12.8%	12.5%	0.21
IVSD ≥ + 3	27.2%	18.8%	**0.01**
PWD ≥ + 3	35.2%	20%	** < 0.001**
LVIDD ≥ + 3	8%	7.5%	0.25
FS ≤ 20%	4.8%	2.5%	0.36

Paired *t*-test, 95% CI *p* < 0.05

*IVSD* interventricular septal thickness in diastole, *LVIDD* left ventricular internal diameter during end-diastole, *PWD* posterior wall thickness in diastole, *LVMI* left ventricular mass index, *RWT* relative wall thickness, *LVEF* left ventricular ejection fraction, *FS* fractional shortening

Values in bold represent statistically significant changes

### Cardiac remodelling

At presentation, only 47 patients (30.9%) had normal cardiac geometry which increased to 63 patients (41.7%) post-intervention. At presentation, 46 patients (30.1%) showed concentric hypertrophy which significantly decreased to 24 patients (15.5%) post-intervention (*p* = 0.03). Further details on cardiac remodelling can be seen in Table 3.

**Table 3:** Proportion of children with cardiac geometry changes pre- and post-intervention

	Normal geometry	Concentric hypertrophy	Eccentric hypertrophy	Concentric remodelling
Pre-intervention	30.9%	30.1%	35.4%	3.5%
Post-intervention	41.7%	15.5%	36.7%	6.3%
Paired *t*-test *p*-value	0.12	**0.03**	0.06	0.36

Paired *t*-test, significance level *p* < 0.05

Value in bold represent statistically significant changes

### Children presenting with heart failure

At our centre, 6 children (3.9%) presented with heart failure, as defined by a severely abnormal FS value (≤ 20%) on their first echocardiogram. All children presenting with heart failure had improved findings in all echocardiographic parameters (IVSD, PWD, LVIDD, LVMI, RWT, LVEF and FS) after intervention. However, one child remained in heart failure and had persistently abnormal echocardiographic parameters, while all other patients with resolving heart failure showed a disappearance of severely abnormal echocardiographic parameters post-intervention. The greatest reduction in number of children with abnormal echocardiographic parameters was seen in PWD in which there was a 60% reduction (5/6 pre-intervention to 2/6 post-intervention). Just one child (16.7%) remained with a severely abnormal FS compared to six pre-intervention.

At presentation, 5 of the children presenting with heart failure had SBP readings above the 99th centile. At last follow-up post-intervention, only one patient had SBP readings above the 99th centile. The number of antihypertensives also reduced post-intervention in these children; at referral, 4 children required 3 or more antihypertensives and post-procedure, only one child required 3 or more antihypertensives and the remaining children either required none or just one antihypertensive agent.

### Surgical intervention

At our centre, 27 children (17.8%) underwent a surgical intervention following at least one endovascular intervention. Surgical interventions included aortic bypass, in situ arterial re-implantation, nephrectomy and auto-transplant. At presentation, 24 of those children (88.8%) had SBP readings above the 95th centile, with 23 of those children (96%) being > 99th. After surgical intervention, the proportion of children with SBP readings above the 95th centile decreased to 7 children (25.9%) (*p* < 0.01).

There was a reduction in the proportion of children with abnormal echocardiographic parameters after at least one surgical and endovascular intervention, and no children had severely abnormal cardiac measurements for IVSD, PWD, LVIDD, LVMI, RWT, FS or LVEF on their last echocardiogram.

At referral, the median number of medications children received was 3.0 (IQR 4.0–2.0), but at last follow-up, this decreased to a mean of 1.4 (SD ± 1.7).

### Regression analysis

Linear regression analysis was carried out to investigate the possibility of a confounding relationship between change in blood pressure centiles and cardiac dimensions. Other covariates included age, presence of MAS, number and type of antihypertensives and number of interventions. This produced a regression model with an *R* value of 0.73 (*p* = 0.001). This analysis found no significant relationship between LVMI and blood pressure; however, it did find that the children with MAS and children undergoing surgical interventions were more strongly associated with a reduction in LVMI post-intervention. Other covariates did not influence change in LVMI post-intervention. Further details can be seen in Table 4.

**Table 4:** Linear regression analysis

	Linear regression coefficient	Two-sided *p*-value
SBP centile post-intervention	− 0.16	0.65
Age	− 1.78	0.23
MAS	− 33.75	**0.03**
Surgical interventions	− 33.89	**0.04**
Total number of interventions	1.53	0.42
Number of antihypertensive medications	− 2.96	0.73
Type of antihypertensive medication	− 5.9	0.73

Association between left ventricular mass index change from pre- to post-intervention, and age, MAS, SBP centile, surgical interventions, total number of interventions, number of antihypertensives and types of antihypertensives post-intervention. Statistically significant associations are given in bold

*SBP* systolic blood pressure, *MAS* middle aortic syndrome

### Glomerular filtration rate

There was a 2.3% increase in mean eGFR across the cohort post-endovascular and/or surgical intervention. While this was not statistically significant, it does indicate the preservation of kidney function post-procedure.

## Discussion

In this single-centre, retrospective, observational study, we looked at whether there is an improvement in cardiac outcomes in children with RenoVH after at least one endovascular intervention and/or surgical intervention. It is the biggest study investigating endovascular and surgical interventions in children with RenoVH and the first to report on the echocardiographic outcomes associated with these treatments. It is the first study to provide data on cardiac function at the time of diagnosis and report on cardiac remodelling post-intervention.

Results show an improvement in echocardiographic outcome after endovascular and/or surgical intervention and that with repeat endovascular intervention, it is possible to achieve regression of previously abnormal cardiac structure and function. Although renovascular disease has significant morbidity and mortality, it is encouraging that these data reflect a positive outlook for patients.

The average age of our cohort was 6 years, similar to that in previous studies which include prepubescent children [8, 9]. The most prevalent syndrome was NF1 which is similar to previous literature [10].

After intervention, the number of hypertensive children decreased by over half, and those with SBP readings above the 99th centile decreased by nearly five-fold. This confirms previous results that interventions are a successful way of decreasing hypertension in children with RenoVH [11–15].

All 152 participants received at least one endovascular intervention making this the largest retrospective cohort study investigating cardiovascular outcomes post-endovascular intervention. Most required only one intervention, similar to other studies [9, 16, 17].

There is no current literature on cardiac structure in children at the time of diagnosis and post-intervention. Our study reports an improvement in average *z*-scores for the considered echocardiographic parameters from baseline to last echocardiographic follow-up (except in LVIDD). Parameters for LVH (PWD, IVSD and LVMI) saw the greatest improvement post-intervention. There were statistically significant differences between first and last echocardiograms in abnormal *z*-scores for PWD and in severely abnormal *z*-scores for IVSD and PWD and a reduction in LVMI. Across the cohort, the greatest reduction in the proportion of children with an abnormal and severely abnormal *z*-score was in PWD and LVMI, further suggesting the cardiac outcome most improved by intervention was LVH.

The cohort of patients who underwent surgery saw the greatest reduction in number of patients with abnormal *z*-scores. Surgical interventions were also strongly associated with an improvement in LVMI on our regression model. These results support our study hypothesis that cardiac parameters would improve with surgical and/or endovascular intervention, but those who undergo surgical interventions do particularly well from a cardiac perspective. Furthermore, our regression model shows that children presenting with MAS are more likely to see an improvement in cardiac outcomes with interventions than those without MAS.

Almost half of the children (*n* = 70, 45.9%) saw a reduction in medication load since referral, indicating the effectiveness of intervention at treating high BP, as already known [15]. Reduced number of medications contributes to a better quality of life and reduces the risk of potential side effects. Furthermore, an easier medication regimen may decrease the risk of non-adherence and, therefore, also of acute hypertensive exacerbation.

In the 6 children who presented with heart failure, our study showed a reduction in the proportion of children with abnormal and severely abnormal echocardiographic values. Average *z*-scores decreased in all parameters. After intervention, only one patient remained with uncontrolled BP. Therefore, our study showed that patients with severe cardiac dysfunction can improve both heart failure and echocardiographic parameters following endovascular and/or surgical intervention.

The proportion of children who underwent a surgical intervention with systolic BP readings above the 95th and 99th centile decreased. Approximately 75% who underwent surgical intervention were no longer hypertensive at last follow-up. The proportion with abnormal echocardiographic parameters reduced, with the greatest improvement seen in LVIDD, suggesting a possible improvement in cardiac filling and contractility. A total of 70.4% reduced their pharmacological load with nearly half of patients (44.4%) stopping all medication by last follow-up. This confirms previous literature, which highlights the interest of a combined treatment approach [18, 19].

There was possibility for bias as confounding variables were not adjusted for in analysis, and thus to minimise investigator bias, we asked four blinded cardiologists to re-measure and re-calculate all echocardiographic parameters and *z*-scores. This was, however, a single-centre observational study whose aim was mainly descriptive. Furthermore, the paired design (with every patient serving as his/her own control, pre-/post-intervention) minimized confounding. Additionally, using US-based *z*-scores may not be appropriate in a multi-ethnic international cohort as described here; however, there are no specific *z*-scores available for every sub-population, so we chose to use the most widely used and accepted *z*-scores. Since this study had a paired, self-control design, this should mitigate some of these effects and should not pose a major impact on these results.

Linear regression analysis was carried out to determine any potential confounding relationship between blood pressure and LVMI. This found that blood pressure centiles did not statistically significantly influence LVMI, indicating that any changes in LVMI post-intervention occurred independently of changes in blood pressure post-intervention. This is a very interesting finding and is not one that has been reported elsewhere in the literature due to the lack of studies looking at cardiovascular outcomes in children with RenoVH. Unfortunately, our data cannot explain this finding, nor can we confirm whether this is also the case for children who only undergo pharmacological management without any endovascular or surgical interventions. This is one of the key reasons that this particular area requires further study, ideally through a multi-centre collaborative study.

We included a long study period, over which time medical practice changed. Nonetheless, this period was needed to allow time for interventions to have impact on disease progression. We did not carry out analyses on those with only endovascular interventions, which made delineating the effectiveness of an individual treatment modality difficult. There have also not been other studies that have looked at children with only endovascular interventions and their cardiovascular outcomes, so it would be difficult to compare such results. However, in clinical practice, most children will have a combined treatment approach (medical management + / − endovascular interventions + / − surgical interventions) and not all patients are suitable for all types of interventions and therefore it is clinically useful to study their joint effectiveness. We also did not carry out analyses on diastolic function as these parameters were not routinely assessed on echocardiograms, but as LVH can cause diastolic dysfunction, it is important that this is investigated in further studies. Additionally, in our study, fractional shortening and ejection fraction were calculated using the Teicholz method, which is not the preferred method, but we did not have all the Simpson’s biplane ejection fractions for all patients as is the recommended calculation.

There is a need for multi-institutional studies to explore the cardiovascular outcomes of intervention compared to medical management alone to investigate the relative efficacy at reducing damage to the cardiovascular system. There are limited studies researching percutaneous transluminal angioplasty in small children with RenoVH [9, 15, 16, 20–23], and thus more research is needed on the outcomes of RenoVH in those under 2 years old.

In conclusion, this study reports improvement in cardiac remodelling after an average of two endovascular interventions with preservation of kidney function. Systolic BP outcomes improved with a reduction in the proportion of patients with readings above the 95th centile. Patients who presented in heart failure saw improved cardiac contractility and reduced LVH which was independent of blood pressure changes. The average number of medications they took also reduced, and pharmacological load post-intervention reduced whereby 32 patients no longer took any medication at last follow-up. This furthers the evidence that even when children present with progressive and life-threatening disease, they can still be effectively managed with endovascular + / − surgical intervention. We hope this research will aid clinical practice to provide evidence that children suffering from RenoVH and its cardiovascular implications can eventually improve with repeated endovascular intervention. Multi-centre collaborative studies are required to further explore the cardiac outcomes, particularly the apparent lack of relationship to blood pressure changes.

### Supplementary Information

Below is the link to the electronic supplementary material.

Graphical abstract (PPTX 46 KB)

Supplementary file2 (DOC 62 KB)

## Data Availability

Anonymised data may be shared on an individual basis if needed. Please contact the corresponding author to arrange this.
