# Supplementary material for: Cardiovascular outcomes improve in children with renovascular hypertension following endovascular and surgical interventions
Source: Pediatr Nephrol. 2023 Sep 2;39(2):521–30. doi: 10.1007/s00467-023-06123-5 (PMC10728245; doi:10.1007/s00467-023-06123-5)
Supplement: Supplementary file 1 — Graphical abstract (PPTX 46 KB) [file 467_2023_6123_MOESM1_ESM.pptx]

## Slide 1
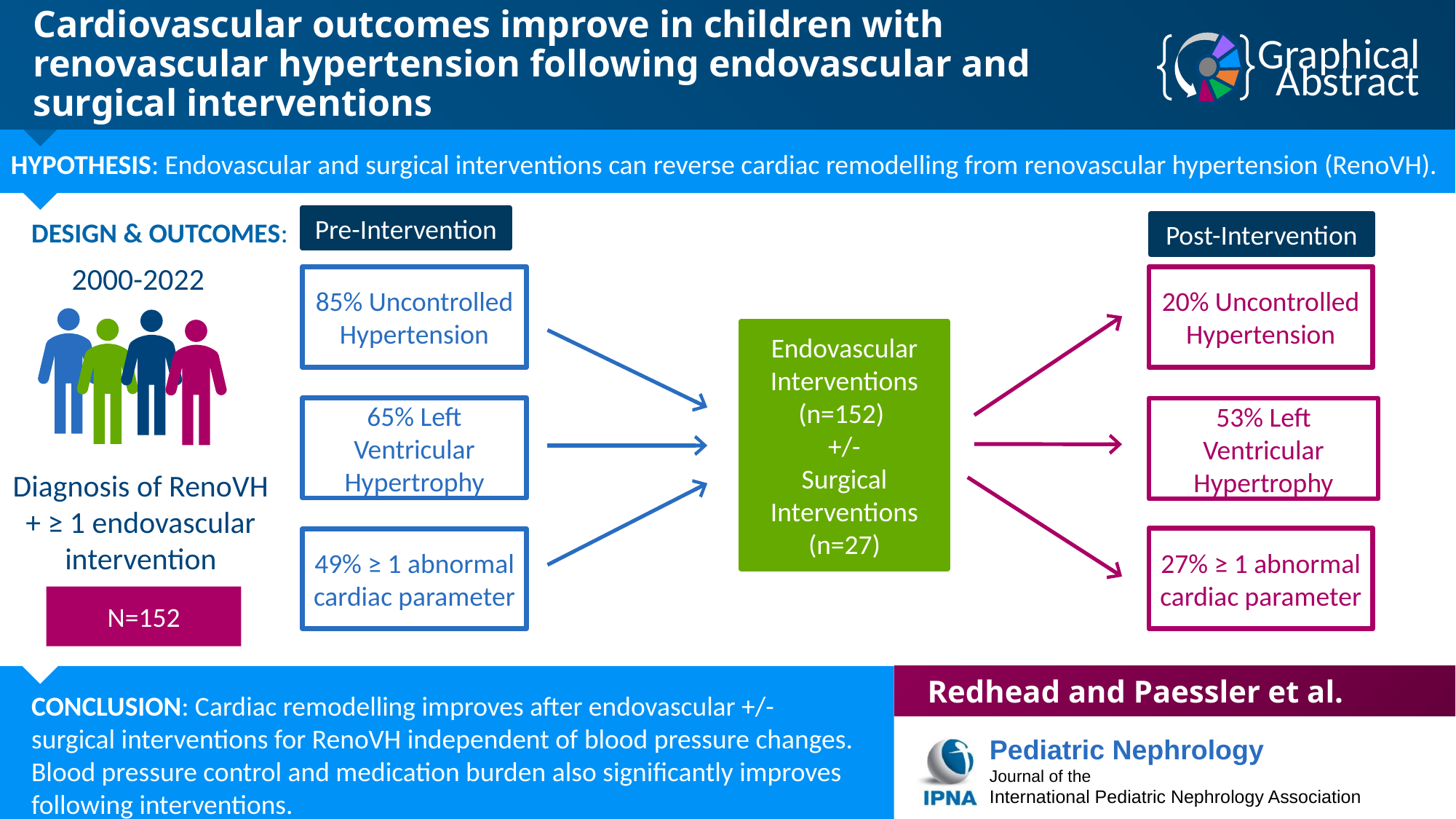

Cardiovascular outcomes improve in children with renovascular hypertension following endovascular and surgical interventions
HYPOTHESIS: Endovascular and surgical interventions can reverse cardiac remodelling from renovascular hypertension (RenoVH).
Pre-Intervention
DESIGN & OUTCOMES:
Post-Intervention
2000-2022
85% Uncontrolled Hypertension
20% Uncontrolled Hypertension
Endovascular Interventions (n=152)
+/-
Surgical Interventions
(n=27)
65% Left Ventricular Hypertrophy
53% Left Ventricular Hypertrophy
Diagnosis of RenoVH + ≥ 1 endovascular intervention
27% ≥ 1 abnormal cardiac parameter
49% ≥ 1 abnormal cardiac parameter
N=152
Redhead and Paessler et al. 2023
CONCLUSION: Cardiac remodelling improves after endovascular +/- surgical interventions for RenoVH independent of blood pressure changes. Blood pressure control and medication burden also significantly improves following interventions.
