# Supplementary material for: Cardiovascular outcomes improve in children with renovascular hypertension following endovascular and surgical interventions
Source: Pediatr Nephrol. 2023 Sep 2;39(2):521–30. doi: 10.1007/s00467-023-06123-5 (PMC10728245; doi:10.1007/s00467-023-06123-5)
Supplement: Supplementary file 2 — Supplementary file2 (DOC 62 KB) [file 467_2023_6123_MOESM2_ESM.doc]

| Variable | | | Frequency (n=152) |
| --- | --- | --- | --- |
| Gender | Male | | 95 (62.5%) |
|  | Female | | 57 (37.5%) |
| Country of origin | UK | | 99 (65.1%) |
|  | Abroad | | 53 (34.9%) |
|  |  | Bahrain | 1 (0.7%) |
|  |  | China | 1 (0.7%) |
|  |  | Cyprus | 1 (0.7%) |
|  |  | Denmark | 9 (5.9%) |
|  |  | Germany | 3 (2%) |
|  |  | Greece | 5 (3.3%) |
|  |  | Holland | 2 (1.3%) |
|  |  | Iran | 1 (0.7%) |
|  |  | Ireland | 7 (4.6%) |
|  |  | Italy | 2 (1.3%) |
|  |  | Kuwait | 2 (1.3%) |
|  |  | Netherlands | 1 (0.7%) |
|  |  | Norway | 2 (1.3%) |
|  |  | Oman | 2 (1.3%) |
|  |  | Portugal | 2 (1.3%) |
|  |  | Qatar | 1 (0.7%) |
|  |  | Saudi Arabia | 3 (2%) |
|  |  | Serbia | 5 (3.3%) |
|  |  | Slovenia | 1 (0.7%) |
|  |  | United Arab Emirates | 2 (1.3%) |
| Syndrome | No syndrome | | 109 (71.7%) |
|  | Underlying syndrome | | 44 (28.3%) |
|  |  | NF1 | 22 (14.5%) |
|  |  | Williams syndrome | 8 (5.3%) |
|  |  | Alagille syndrome | 4 (2.6%) |
|  |  | Cutis marmorata telangiectatica congenita | 2 (1.3%) |
|  |  | NF2 | 1 (0.7%) |
|  |  | Elastin Gene Deletion | 1 (0.7%) |
|  |  | Adams Oliver syndrome | 1 (0.7%) |
|  |  | Phacomatosis pigmentokeratotica | 1 (0.7%) |
|  |  | Treacher Collins syndrome | 1 (0.7%) |
|  |  | Generalised arterial calcification of infancy | 1 (0.7%) |
|  |  | Fragile X syndrome | 1 (0.7%) |
| MAS | No | | 93 (61.2%) |
|  | Yes | | 59 (38.8%) |
| Moyamoya disease | No | | 149 (98%) |
|  | Yes | | 3 (2%) |
| Congenitally absent kidney | No | | 150 (98.7%) |
|  | Yes | | 2 (1.3%) |
|  |  | Right | 1 |
|  |  | Left | 1 |

*Supplementary File 1: Contingency table displaying the baseline characteristics of the study population.*
